# A 3D millifluidic model of a dermal perivascular microenvironment on a chip†

**DOI:** 10.1039/d4lc00898g

**Published:** 2025-01-06

**Authors:** Chiara Martinelli, Alberto Bocconi, Sofia Milone, Teresa Baldissera, Leonardo Cherubin, Giovanni Buccioli, Simone Perottoni, Claudio Conci, Giulio Cerullo, Roberto Osellame, Giuseppe Chirico, Emanuela Jacchetti, Manuela Teresa Raimondi

**Affiliations:** a Department of Chemistry, Materials and Chemical Engineering “Giulio Natta”, Politecnico di Milano Piazza L. da Vinci, 32 20133 Milan Italy chiara.martinelli@polimi.it alberto.bocconi@polimi.it sofia.milone@mail.polimi.it teresa.baldissera@mail.polimi.it leonardo.cherubin@polimi.it giovanni.buccioli@polimi.it simone.perottoni@polimi.it claudio.conci@polimi.it emanuela.jacchetti@polimi.it manuela.raimondi@polimi.it; b Institute for Photonics and Nanotechnologies (IFN), CNR and Department of Physics, Politecnico di Milano Piazza L. da Vinci 32 20133 Milan Italy giulio.cerullo@polimi.it roberto.osellame@cnr.it; c Department of Physics, Università di Milano-Bicocca Piazza della Scienza, 3 20126 Milan Italy giuseppe.chirico@unimib.it

## Abstract

The process of angiogenesis plays a pivotal role in skin regeneration, ensuring the provision of nutrients and oxygen to the nascent tissue, thanks to the formation of novel microvascular networks supporting functional tissue regeneration. Unfortunately, most of the current therapeutic approaches for skin regeneration lack vascularization, required to promote effective angiogenesis. Thus, *in vitro* tridimensional models, complemented with specific biochemical signals, can be a valuable tool to unravel the neovascularization mechanisms and develop novel clinical strategies. In this work, we designed and validated a tridimensional microstructured dynamic model of the dermal perivascular microenvironment on a chip. We carried out the fabrication of an array of microstructures by two-photon laser polymerization, then used as a 3D substrate for co-culture of human dermal fibroblasts and endothelial cells. We included the substrate in a miniaturized optically accessible bioreactor (MOAB) which provides the physiological interstitial flow, upon perfusion in the presence or absence of the pro-angiogenic stimuli VEGF and TGF-β1. We determined the parameters to be applied under dynamic conditions by an *in silico* model simulating individual 3D microenvironments within the bioreactor's chambers. We computed the fluid velocity and wall shear stress acting on endothelial cells along with the oxygen concentration profile, and we chose the most suitable flow rate for maintaining dermal physiological conditions. Experimental results showed the effectiveness of the developed platform as a 3D dynamic model of angiogenesis. This is the first combined experimental and computational study involving chemically stimulated 3D co-cultures for successfully simulating the physiological dermal perivascular microenvironment.

## Abstract 2

A dermal perivascular microenvironment has been reproduced on a chip within the MOAB's chambers by combining 3D microscaffolds, and controlled fluid dynamics and mass transport conditions, predicted by computational modelling of *in vivo* angiogenesis.

## Graphical abstract

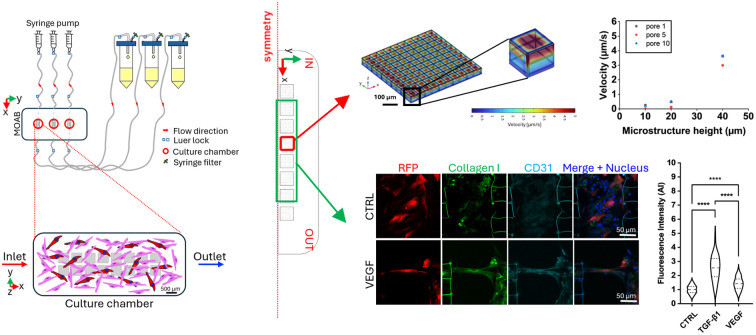

## Introduction

Intact healthy skin is a highly adaptive, multifunctional organ that is of paramount importance, governing the overall bodily homeostasis.^1^ Any disruption to its integrity compromises its functions and leads to wound formation. Skin wounds are a prevalent occurrence during human life.^2^ Any altered step of the healing cascade, from hemostasis to inflammation, proliferation and remodeling, contributes to the formation of hypertrophic scars, peculiar to the chronicity of wounds, mainly characterized by delayed re-epithelialization and reduced angiogenesis.^3^ Chronic wounds cover a large portion of healthcare costs and most of the current treatments are only moderately successful, leading to a pressing need for new and more effective therapies.^4^ Drug delivery systems, cell sheets, injectable hydrogels with or without cells and the fabrication of skin substitutes *via* bio-scaffold production represent promising approaches but present the challenge of high costs.^5,6^ Consequently, greater emphasis should be put on investigating the cellular and molecular components involved in the wound healing process, with the final aim of developing novel and more cost-effective therapeutic strategies. During wound healing, angiogenesis in the dermal layer plays a pivotal role in providing oxygen and nutrient supply to the regenerating tissue. Thus, mimicking this process *in vitro* is of key importance for analyzing in depth the phenomenon and consequently proposing new effective strategies to treat it. In the field of skin engineering, the vascularization of three-dimensional (3D) constructs is an essential requirement for achieving the best resemblance to the physiological perivascular microenvironment, while improving proper *in vivo* integration. Currently, the major obstacle to the clinical application of tissue engineered scaffolds remains the lack of adequate vascular supply, limiting their physiological function and often resulting in cell necrosis.^7^ During wound healing, quiescent vessels are exposed to proangiogenic factors, prompting neighboring endothelial cells to differentiate into stalk cells that proliferate, migrate specializing in tip cells, and culminate in the formation of sprouting vessels, capable of fusing together, re-establishing the blood flow.^8^ Angiogenesis is modulated by several chemical factors, but also involves complex interactions between vascular cells and the 3D extracellular environment.^9^ Different experimental strategies have been adopted for the promotion of angiogenesis *in vitro*, but most of them do not properly model the *in vivo* microenvironment, including tridimensionality, perfusion and the presence of suitable biological elements. Among them, the main chemical regulator is vascular endothelial growth factor (VEGF), a proangiogenic factor stimulating endothelial cells to proliferate, migrate, differentiate and survive, allowing new blood vessel formation. Cutaneous wounds are characterized by high levels of VEGF, synthesized by multiple cell types, including keratinocytes, macrophages and fibroblasts in response to injury.^10^ Another crucial molecule is transforming growth factor-beta (TGF-β), which exerts a significant influence on inflammation, angiogenesis, epithelialization and connective tissue regeneration and is secreted by macrophages, fibroblasts, platelets and keratinocytes. Most of the early studies performed to understand the angiogenesis process relied on the implementation of 2D models based on co-culture of endothelial cells and fibroblasts, administered with VEGF and TGF-β for prompting the creation of a favorable cellular microenvironment.^11–15^ Although 2D *in vitro* models can be used to investigate key behaviors of endothelial cells, such as migration, proliferation and organization among other supportive cells, they currently cannot mimic some typical tridimensional features, such as lumen formation and differentiation into stalk and tip cells.^15^ Indeed, both a 3D microenvironment and the set-up of perfusable systems able to mimic the flow conditions to which cells are physiologically subjected are essential for obtaining reliable *in vitro* models. In the context of mimicking the 3D microenvironment fundamental for providing support to microvasculature, the presence of a fabricated collagen biopolymer and of HUVEC, combined with fibroblasts and keratinocytes, developed a well-ordered vascular network. This highlighted the need for a 3D substrate and multiple cell types for obtaining an endothelialized skin equivalent.^16^ However, although successful, this and similar systems lack the crucial stimulus provided *in vivo* by the presence of tissue interstitial flow that takes part in regulating blood capillary morphogenesis and endothelial cell morphology.^17^ Perfusion is fundamental for achieving efficient molecule transport and physiological functioning of *in vitro* tissues, and it is generally obtained by means of bioreactors.^18^ Recently, microfluidic technologies have combined tridimensionality and dynamic cell culture conditions in *in vitro* models, paving the way to better replicate the angiogenesis mechanisms.^19–23^ Vickerman *et al.* developed a microfluidic device for 3D cell culture to study capillary morphogenesis of human adult dermal microvascular endothelial cells (HMVEC-ad) cultured in medium enriched with VEGF and seeded into an injectable biocompatible scaffold obtaining complex interconnected multicellular capillary-like structures.^20^ In an another study, Douglas *et al.* investigated microvascular networks containing RFP-labeled HUVEC and fibroblasts co-cultured for 7 days in fibrin gels, conditioned by a custom pump system imposing a shear stress of about 0.5 Pa, and demonstrated that fibroblasts wrapped around vessels, and synthesized collagen I, highly co-localized with vessels.^24^ Thus, fibroblasts and their products can constitute an *in vitro* extracellular matrix (ECM) environment that surrounds endothelial cells and supports them in forming capillary-like structures. Similar outputs were obtained using a perfusable model, consisting of HUVEC and human lung fibroblasts (HLF) at a seeding ratio of 5 : 1 in the presence of VEGF, in which connected structures consistent with vascular networks were generated and quantified in terms of length and diameter.^25^ Promising results were obtained by seeding cells embedded into collagen and fibrin gels in microfluidic channels, allowing the formation of tubes and networks upon perfusion.^26–28^ However, the use of straight microfluidic channels with rectangular cross sections remains distant from the *in vivo* physiological microvascular beds.^26^ Shirure *et al.* developed an *in vitro* microfluidic platform to stimulate 3D angiogenesis of endothelial cells and stromal fibroblasts seeded in a fibrin extracellular matrix.^29^ They demonstrated that, while angiogenesis can be guided or directed by interstitial flow (0.1–10 μm s^−1^) alone, endothelial cells must be in an active state (in the presence of VEGF) to be effectively responsive to fluid flow.^29^ Recently, biomimetic skin-on-chip models have been introduced co-culturing different cell types in 3D structures, mimicking the dermal ECM and perfused to recreate the physiological flow-induced shear stress, necessary for stimulating endothelial cell organization into tubular-like structures. For instance, Kim *et al.* employed 3D bioprinting for engineering a complex 3D human skin model, even though it was devoid of a suitable vasculature, needed for providing physiological oxygen and nutrient supply.^30^ From the reported research, it can be inferred that, although many systems have been implemented to reconstitute 3D microenvironments equipped with perfusion and effectively mimicking the processes of angiogenesis and skin regeneration, chemical, physical and biological factors have been rarely integrated into the same experimental setup. Moreover, experimental data analysis often needs supporting models to better interpret the obtained results; therefore combining the experimental and computational approaches may enable better comprehension of the *in vitro* angiogenesis process.^31^ The combination of computational and experimental studies can provide efficient pipelines to thoroughly analyze biological processes, while minimizing animal experiments, which are still frequently involved in the field of wound healing research. Several mathematical models have been optimized to mimic angiogenesis in wound healing; however, they mainly make use of 1D or 2D continuum domains, while biological processes occur in 3D. Recently, some computational analyses have been published aimed at finely tuning the fluid dynamics parameters within microfluidic platforms, and specifically in niches-on-a-chip models.^32,33^ For instance, Perottoni *et al.* employed the miniaturized optically accessible bioreactor (MOAB) (MOAB S.r.l., Milan, Italy), a multi-chamber millifluidic culture system, able to mimic the microenvironment of mesenchymal stem cells (MSCs) near blood capillaries and within the stem cell niche.^33^ They validated this miniaturized platform for profiling stem cell metabolism, specifically allowing tunable and measurable oxygen tension gradients that impact their metabolism in monolayer culture. Another study by our group predicted the fluid dynamics to which mesenchymal stem cells are subjected during perfused culture in 3D microscaffolds.^34^ We found that the lower values predicted near the bottom of the microscaffold corresponded well with physiologically relevant levels of interstitial fluid velocity (of average 0.6 μm s^−1^, up to 2 μm s^−1^) measured *in situ* in tissue.

**Fig. 1:**
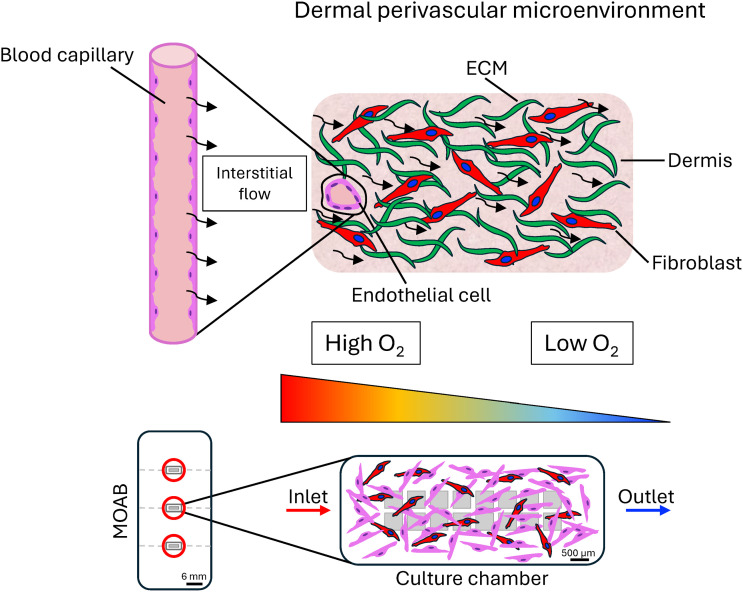
Dermal perivascular microenvironment on a chip. At the top, a graphical representation of the *in vivo* dermal perivascular microenvironment is shown. Dermal fibroblasts constitute an intricate ECM network, supporting endothelial cells that constitute a blood capillary providing oxygen to the surrounding dermis. The interstitial flow and oxygen concentration gradient (triangle with color scale) are responsible for providing mechanical and chemical stimuli to the tissue. At the bottom, this model is experimentally reproduced *in vitro* within the miniaturized optically accessible bioreactor's (MOAB) chambers by combining the use of 3D cell culture microscaffolds, and controlled physiological fluid dynamics and mass transport conditions, predicted by finite element computational modelling, mimicking those occurring during *in vivo* angiogenesis within the dermis.

In the present work, we report a significant advancement to the concept of modelling a controlled physiological dermal perivascular microenvironment. We designed a novel 3D scaffold platform to fit the MOAB's chambers, and we optimized the fluid dynamics and mass transport parameters to simulate specific 3D microenvironments with varying levels of oxygen tension, to model in a realistic way the angiogenic processes occurring *in vivo* (Fig. 1). Here we describe the details of the development of this novel 3D dynamic model of angiogenesis in a dermal-like environment, able to mimic an independent physiological dermal perivascular microenvironment located far from the terminal arterioles. A robust *in silico* model was capable of predicting the fluid dynamics in the microenvironments, with the derived velocities and shear stresses acting on endothelial cells, generated by perfusion, and crucial for replicating the *in vivo* field variables of dermal interstitial flow in the cell-populated microenvironments. Moreover, we investigated the oxygen concentration profile, and we chose the best flow rate to avoid hypoxia while maintaining the dermal physiological oxygen concentration. We fabricated the 3D highly porous microstructures by two-photon polymerization (2PP), we seeded the microscaffolds with co-cultures of human dermal fibroblasts and endothelial cells, and we stimulated cells with the pro-angiogenic factors VEGF and TGF-β1. The 3D constructs were then inserted into the bioreactor, where physical and chemical cues were coupled by perfusing the system with growth factors' enriched culture medium. Results of the experiments performed under static and dynamic conditions were compared in terms of cell morphology and organization, showing that the successful formation of a primordial microvascular network surrounded by synthesized collagen I can be obtained only in the presence of the 3D microstructures combined with dynamic culture conditions and VEGF administration.

## Results and discussion

### Fabrication of 3D microstructures and MOAB assembly

**Fig. 2:**
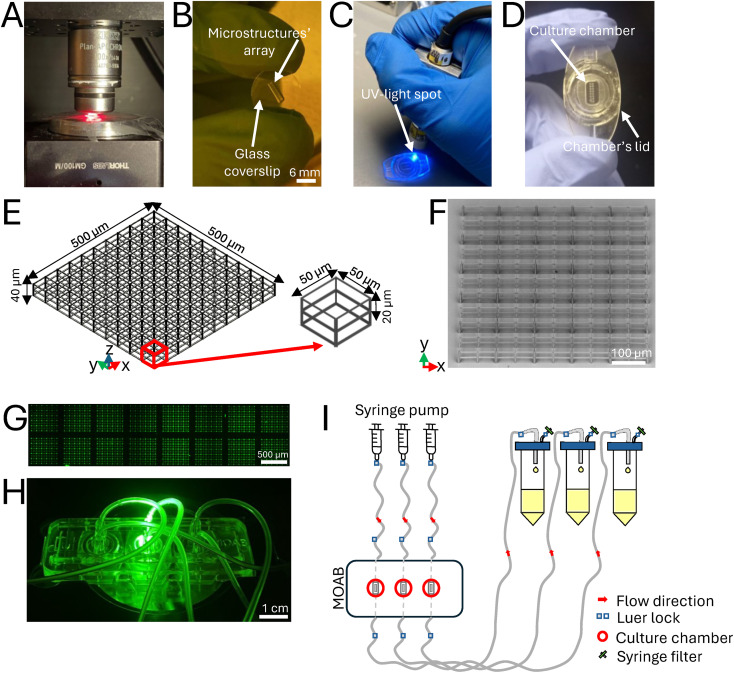
Experimental setup. A) Sample holder with a red-light emitting diode used for sample visualization during the writing procedure. B) Microstructures' array fabricated on the glass coverslip, visible after the development procedure. C) UV-light spot, used to cure a biocompatible glue, and visible during the sealing procedure of the glass coverslip to the chamber's lid. D) Microstructures' array included in the bioreactor's chamber inside a lid. E) CAD model of one microstructure composing the array, measuring 500 × 500 × 40 μm^3^. Design of the elementary unit (red arrow) constituted by two pores, each measuring 50 × 50 × 20 μm^3^. F) SEM image of one microstructure. G) Confocal laser scanning microscopy image of the entire microstructures' array. H) MOAB assembled, positioned on an optical microscope (Olympus IX70, Japan) and illuminated by a fluorescent mercury lamp filtered by a Semrock BrightLine TRITC-A fluorescent filter (Olympus, Japan). I) Scheme of the assembled hydraulic circuit: a high precision syringe pump was used to independently perfuse the three chambers of the bioreactor. At the outlet three reservoirs collected the waste products.

The MOAB device is a multi-chamber bioreactor constituted by medical grade polycarbonate, presenting three magnetically lockable chambers with lids hosting a rectangular glass surface base (3 mm × 6 mm × 0.5 mm, volume = 9 μl) for cell seeding and live imaging.^35^ In this work, the MOAB was employed for interstitial flow perfusion of co-cultures seeded in the 3D microenvironment. The three chambers were perfused independently for simultaneously testing three different experimental conditions (untreated control, TGF-β1 administration, VEGF administration). 3D microstructures were fabricated by 2PP of the biocompatible photoresist SZ2080. Upon drop casting of the resin, the glass coverslip was fixed on an aluminum holder screwed to a gimbal mechanical system for sample alignment; a femtosecond laser was then focused inside the resin to trigger the nonlinear absorption process that induces the highly localized resin polymerization. The sample is then translated with respect to the laser beam to define the three-dimensional structures. To precisely inspect the sample during the writing procedure, a 630 nm emitting diode, placed under the sample holder, was switched on (Fig. 2A) and a CMOS camera was placed on the top of the sample holder to collect the transmitted light. After the fabrication procedure, the microstructures' array was developed to remove the unpolymerized photoresist from the glass coverslip (Fig. 2B), before gluing it to the bioreactor. The glass coverslips covered with the microfabricated scaffolds were placed into the bioreactor's chamber inside each chamber's lid by following a sealing protocol with a biocompatible UV-curable glue (Fig. 2C). The 3D microstructures were arranged in a 2 × 8 matrix to facilitate the evaluation of interstitial flow throughout the tissue depth (Fig. 2D). Each microstructure was characterized by pores of 50 × 50 × 20 μm^3^ size, as previous studies have demonstrated that this pore size facilitates revascularization (Fig. 2E).^36^ The obtained beams have an ellipsoidal shape with an axial dimension of 4.9 ± 0.61 μm and a lateral one of 1 ± 0.1 μm. Scanning electron microscopy (SEM) images were acquired to evaluate the quality of the fabricated microstructures, before sample sterilization and use (Fig. 2F). The entire microstructures' array was imaged by confocal laser scanning microscopy (Fig. 2G) and the MOAB was assembled to a hydraulic circuit that was optimized for perfusing the co-cultures in the three independent optically accessible chambers (Fig. 2H), thanks to the use of a high precision syringe pump. Each circuit was composed of a syringe filled with cell culture medium and a silicon tubing system connected to the inlet and the outlet of each chamber. The lid of each reservoir presented two outlets for i) a tube and ii) a sterile filter, applied to avoid contamination and to balance the internal–external atmospheric pressure difference. At the outlet, three reservoirs collected the waste products (Fig. 2I).

### Computational fluid dynamics analysis

**Fig. 3:**
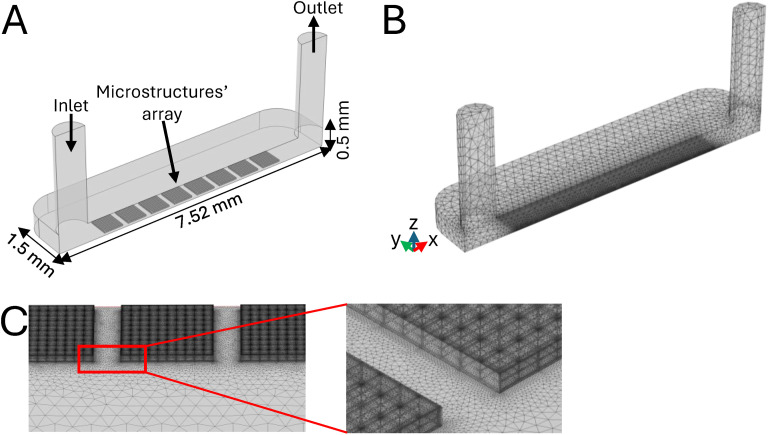
Computational model setup. A) CAD of the MOAB chamber's geometry, indicating the positioning of the inlet, outlet and microstructures' array. B) Tetrahedral mesh used for CFD studies, indicating the finer mesh of the microstructure's elements as compared to the ones composing the chamber. C) Insight of the finer mesh composing the microstructures.

Currently, most of the reported angiogenesis experimental models are based on the fabrication of microfluidic poly(dimethylsiloxane) (PDMS) channels. These aim to induce cell orientation according to a predefined direction and are generally stimulated by the addition of VEGF into the cell culture medium.^19,20,29^ However, these devices present a key limitation: the absence of a 3D support for the cells, thus lacking an essential feature to model the *in vivo* microenvironment. Moreover, many of the *in vitro* angiogenesis assays carried out so far do not present a perfusion system providing an interstitial flow to the cells inside the microstructures, as a physical stimulus required for proper induction of the process.^13,37,38^ In this work, computational fluid dynamics (CFD) simulations were performed to design a reliable model of the 3D dermal perivascular microenvironment. The parameters predicted by our *in silico* model allowed us to develop a suitable device for mimicking *in vitro* the physiological conditions promoting angiogenesis.^18,29^ The longitudinal symmetry of the MOAB's cell culture chamber was exploited to reduce the computational load of the simulations. Therefore, our model replicated a complete chamber with dimensions of 6 mm × 3 mm × 0.5 mm, containing a double linear array of 8 equally spaced microstructures. To reduce computational time, half of the chamber was simulated only, by applying a symmetry condition, resulting in a modeled volume of 6 mm × 1.5 mm × 0.5 mm. The inlet and outlet tubings were modeled as two rigid cylinders (Fig. 3A). A free tetrahedral mesh was built to perform the simulations, setting an element size ranging from 0.1–1.4 mm (Fig. 3B) with a consequent element refinement at the boundaries and edges of the microstructures, as shown in Fig. 3C. Additional simplifications were applied to the experimental model as follows: i) a single cell type, HUVEC, was simulated, as hypothesized to predominantly drive the microvascular network formation and thus comprising 80% of the co-cultured cells within the bioreactor's chamber (cell seeding in a 5 : 1 ratio, HUVEC : RFP-HDF). Moreover, this cell type exhibits the highest metabolic demand. Our model was designed to simulate the most critical conditions possible, ii) only a single growth factor, *i.e.* VEGF, was modeled, as it specifically stimulates endothelial cells and is responsible for angiogenesis both *in vitro* and *in vivo*,^39,40^ iii) the value of oxygen consumption by endothelial cells was obtained from the literature^41^ and used here, as it represents the highest reported value, thereby simulating the most critical conditions. The computational study allowed us to predict the most appropriate parameters for performing co-culture experiments under dynamic conditions. The first one was the flow rate (*Q*) at which the employed syringe pump had to work for providing i) an interstitial flow able to maintain physiological velocities, ii) a wall shear stress within specific target ranges necessary for promoting endothelial cell sprouting,^42,43^ and iii) the oxygen consumption required to avoid hypoxic conditions and, conversely, to accurately mimic the *in vivo* oxygen partial pressure conditions, typically found in dermal tissue.

### Velocity and wall shear stress analyses

**Fig. 4:**
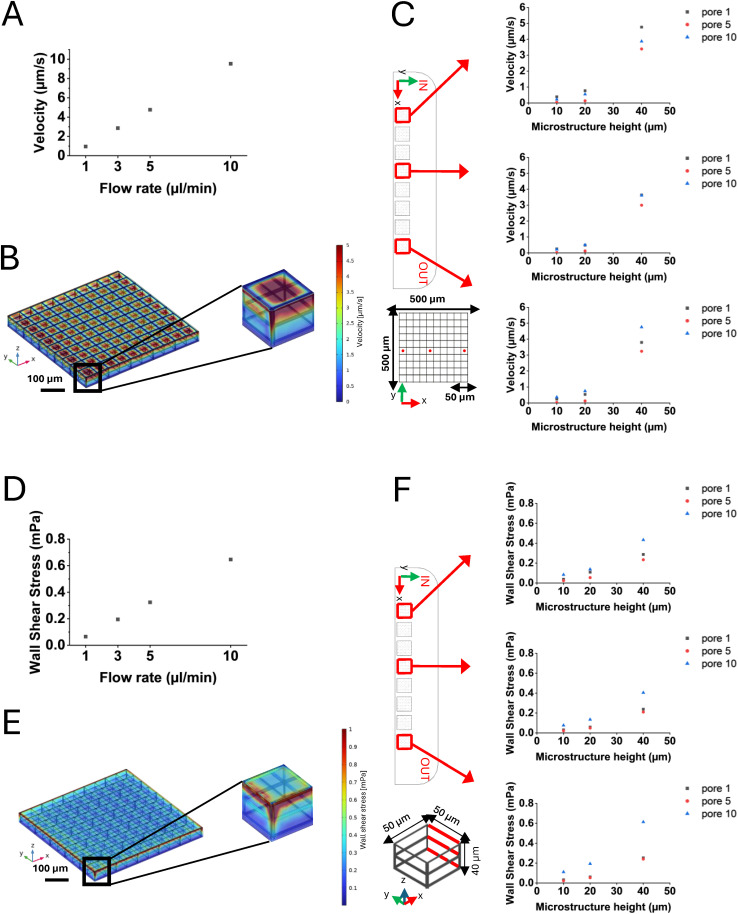
Velocity and wall shear stress predictions. A) Plot of the maximum velocities extracted from the model simulations for the flow rates tested (1,3,5,10 μL min^−1^). Maximum velocity values, predicted at 40 μm height, were analyzed to choose the most suitable flow rate, according to the selected target range. B) Color map of the velocity obtained at 5 μL min^−1^. The highest velocities were reached on top of the first microstructure of the array at the inlet of the chamber. C) Top view of the MOAB's chamber, showing the microstructures where the velocity measurements were performed (red squares), and CAD model of the pores where velocity was measured (red dots). Plots represent punctual velocities predicted in the center of each pore considered, at 10, 20, and 40 μm height. D) Plot of the maximum wall shear stress obtained at the flow rates tested (1,3,5,10 μL min^−1^). Wall shear stress values predicted at 40 μm height were averaged and analyzed to choose the most suitable flow rate, according to the selected target range. E) Color map of the wall shear stress obtained at 5 μL min^−1^. An insight on the first microstructure of the array, at the chamber's inlet, shows the microstructure's edges where the highest values of wall shear stress acted. F) Top view of the MOAB's chamber, showing the microstructures where the wall shear stress measurements were performed (red squares), and CAD model of the pore where the wall shear stress was measured (red bars), corresponding to 10, 20 and 40 μm height. Plots represent wall shear stress averaged values obtained along the three beams, on the side towards the outlet, of each pore considered.

Different cell culture medium flow rates (1, 3, 5, and 10 μL min^−1^) were applied and fluid velocity and wall shear stress were predicted both at the top layer (40 μm) and within the microstructures, to determine the optimal rate for replicating *in vivo* interstitial flow. Fluid velocity ranges established by physiological interstitial flow during angiogenesis have been previously reported.^42,43^ To simulate the most critical scenario for cells, the more restrictive range of 0.1–5 μm s^−1^ was chosen for flow rate analysis.^43^ Examining the top layer of the first and the last scaffolds (located near the inlet and outlet, respectively) we observed that at the predicted flow rate of 10 μL min^−1^, the fluid velocity exceeded the identified physiological range (Fig. 4A). Consequently, the system was examined at the maximum identified flow rate (5 μL min^−1^), within the pores, where, as expected, the highest velocity values were found at the top layer of the structures. The first microstructure of the array, at the chamber's inlet and subjected to the highest stress, is shown, with predicted velocity values ranging from 0.1 to 5 μm s^−1^. The detailed distribution of these velocities can be observed in the color map of Fig. 4B. Additionally, punctual velocity predictions were extracted at heights of 10 μm – corresponding to the height of cells at the chamber's bottom –, 20 μm, and 40 μm from the chamber's bottom layer, specifically at the center of the first, fifth, and tenth pores within the microstructures positioned in the proximity of the inlet, in the chamber center, and near the outlet, to assess variations (Fig. 4C). Regarding the flow rate, the physiological range for the wall shear stress was established based on literature values and corresponding to a range of 0.1–10 mPa.^44^ We simulated the systems across all the designed flow rates (1,3,5,10 μL min^−1^) and we predicted the wall shear stress in the worst-case scenario, focusing on the outer top edges of the first and the eighth microstructures, located near the chamber's inlet and outlet, respectively. All the flow rates produced shear values predicted within the selected range, except for the 1 μL min^−1^ case (Fig. 4D), which did not reach the minimum threshold, and we supposed not sufficient to adequately stimulate the cells. Based on these results, we chose a flow rate of *Q* = 5 μL min^−1^ for our experiments, as it provided a shear stress level comparable to those reported in other studies in perfused cell cultures *in vitro*.^32,33,43^ The detailed distribution of these values can be observed in the color map of Fig. 4E. The wall shear stress values were averaged along three beams, on the side of the microstructure towards the outlet, of each pore considered (the first, the fifth and the tenth of the same microstructures assessed for the velocity analysis), at 10, 20 and 40 μm height (Fig. 4F) to evaluate the variation along the *Z* axis. It is worth noting that, in this work, we focused on calibrating our model of 3D co-culture as close as possible to conditions occurring *in vivo*, specifically addressing the 3D microstructures. Nevertheless, we performed additional computational analyses to evaluate if the array of microstructures could influence the fluid dynamics within the bioreactor's chambers. The wall shear stress predicted and averaged within a totally flat MOAB chamber corresponded to the one predicted in the flat area surrounding the array of microstructures (nearly 0.46 mPa, Fig. S1†). We can conclude that the microstructures' array did not perturb the fluid dynamics inside the chamber and that a 3D microfabricated chamber's lid simultaneously allowed the analysis of cellular behavior on flat surfaces (on the area surrounding the microstructures' array) and in 3D microscaffolds, avoiding the use of entirely flat substrates as controls for co-culture under dynamic conditions.

### Oxygen consumption

**Fig. 5:**
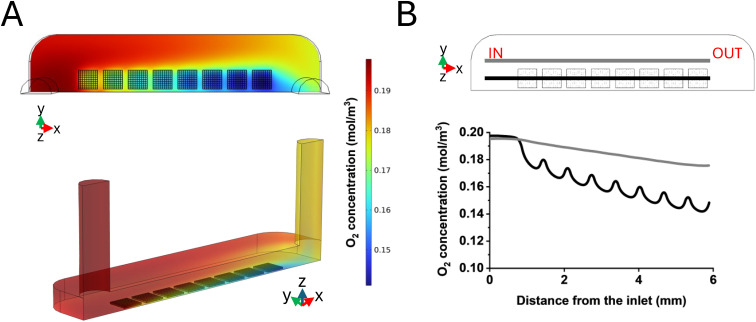
Oxygen consumption derived from computational modelling. A) Color maps of the oxygen concentration predicted on a horizontal section taken at 10 μm height from the bottom surface of the chamber in a microfabricated chamber's lid in the *XY* plane (above) and oxygen concentration plotted on the entire culture chamber (below). B) Plot of the oxygen concentration profile along the MOAB's chamber obtained by imposing a 5 μL min^−1^ flow rate. The grey line indicates measurements performed in flat areas of the chamber surrounding the microstructures; the black line indicates measurements performed inside the microstructures' array.

To avoid a possible condition of hypoxia inside the culture chambers, we simulated endothelial cells' oxygen consumption by setting a flow rate of 5 μL min^−1^, according to the previous numerical outcomes. A concentration of 1% of atmospheric oxygen partial pressure was reported to be the threshold that defines hypoxia for HUVEC.^45^ A normoxia state in accordance with the dermis under physiological conditions, which is reported to correspond to 13% of the atmospheric oxygen partial pressure, was considered.^41^ The concentration profile along the chamber length and the consequent oxygen drop were then predicted, starting from an initial concentration of 0.2 mol m^−3^, according to Henry's law and to the oxygen solubility in the cell culture medium. The predictions were extracted from the computational simulations i) along a reference line crossing the microstructures along the *X* axis. In this direction, most of the cells were supposed to be adherently attached to the substrate, and additionally the medium flow was subjected to a slow down due to the presence of the array, ii) in the flat portion of the chamber on the side of the microstructures. In particular, the reference line was built at a height of 10 μm from the bottom of the chamber, in correspondence to the lowest oxygen level. The color maps of the whole chamber referring to the oxygen concentration predicted on a horizontal section taken at 10 μm in height from the chamber bottom, where 50.000 cells were adhered to the microstructured chamber's lid, are reported in Fig. 5A. In Fig. 5B, a plot of the oxygen concentration profile is reported: a 5.6% decrease in the oxygen concentration was predicted in the presence of the microstructures (black line), which we attributed to the presence of 32.000 cells within the scaffolds, resulting in a final oxygen concentration in the proximity of the outlet of approximately 0.14 mol m^−3^, with respect to the normoxia condition of the dermis. In the flat area alongside the microstructures (grey line), 18.000 adhered cells were considered, and we predicted that cells produced a drop of 2% in oxygen concentration. In conclusion, the predicted oxygen levels were far below from the values of dermis hypoxia (1%).^45^

### Co-culture of endothelial cells and fibroblasts under static and dynamic conditions

**Fig. 6:**
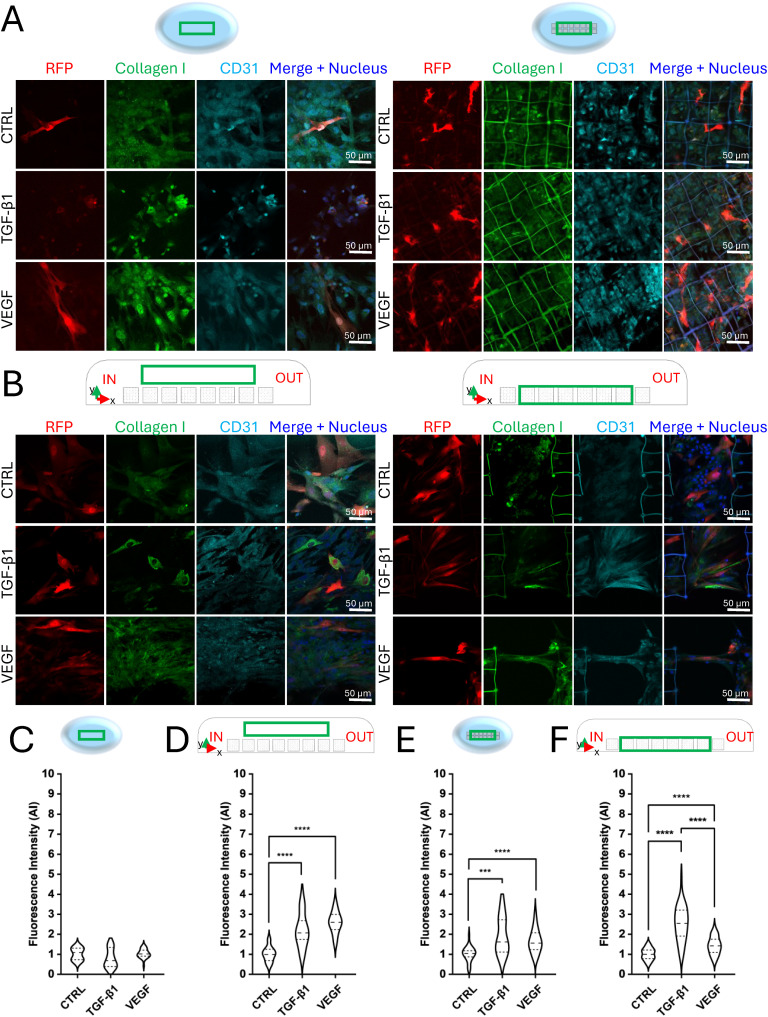
Immunofluorescence and collagen I fluorescence intensity quantification of static and dynamic co-cultures at day 7. A) Confocal images of the stained static co-cultures. Flat substrate on the left, microstructures on the right. RFP, collagen I, and CD31 are visible in red, green and cyan, respectively. B) Confocal images of the stained dynamic co-culture. Flat area on the side of the microstructures' array on the left, microstructures on the right. RFP, collagen I and CD31 are visible in red, green and cyan, respectively. C) Quantification of collagen I synthesized by RFP-HDF under treated and untreated conditions, on flat substrates in static experiments. Data are normalized with respect to the control (0.1% BSA), *n* ≥ 27 for each condition. D) Quantification of collagen I synthesized by RFP-HDF at day 7 in the flat area surrounding the microstructures in dynamic experiments, in the presence of VEGF or TGF-β1. Data are normalized respect to the control (0.1% BSA), *n* ≥ 27 for each condition. ****p-value < 0.0001. E) Quantification of collagen I synthesized by RFP-HDF at day 7 on 3D substrates in static experiments, in the presence of VEGF or TGF-β1. Data are normalized respect to the control (0.1% BSA), *n* ≥ 27 for each condition. ***p-value < 0.001; ****p-value < 0.0001. F) Quantification of collagen I synthesized by RFP-HDF at day 7 on 3D substrates in dynamic experiments, in the presence of VEGF or TGF-β1. Data are normalized respect to the control (0.1% BSA), *n* ≥ 27 for each condition. ****p-value < 0.0001. The green box in the graphical sketches (not scaled) above images indicates regions of the samples where acquisitions were performed.

**Fig. 7:**
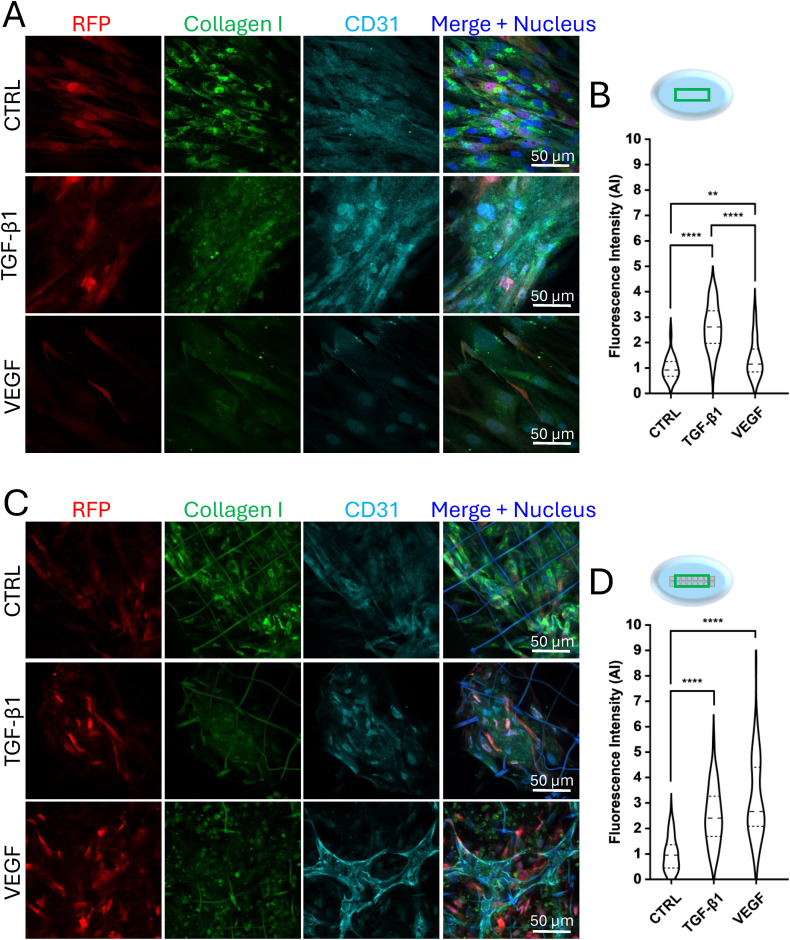
Immunofluorescence and collagen I fluorescence intensity quantification of static co-cultures at day 13. A) Confocal images of the stained static co-cultures at day 13 on a flat substrate. RFP, collagen I and CD31 are visible in red, green and cyan, respectively. B) Quantification of collagen I synthesized by RFP-HDF at day 13 on flat substrates in static experiments, in the presence of VEGF or TGF-β1. Data are normalized respect to the control (0.1% BSA), *n* ≥ 27 for each condition tested. **p-value < 0.01; ****p-value < 0.0001. C) Confocal images of the stained static co-cultures on microstructures on day 13. RFP, collagen I and CD31 are visible in red, green and cyan, respectively. D) Quantification of collagen I synthesized by RFP-HDF at day 13 in the microstructures in static experiments, in the presence of VEGF or TGF-β1. Data are normalized respect to the control (0.1% BSA), *n* ≥ 27 for each condition tested. ****p-value < 0.0001. The green box in the graphical sketches (not scaled) above images indicates regions of the samples where acquisitions were performed.

We performed static co-cultures to investigate cell organization on a flat substrate and 3D microstructures' array and investigated the contribution of VEGF and TGF-β1 stimulation under different environmental conditions. A co-culture of HUVEC and RFP-HDF (relative density 5 : 1) was seeded on a flat substrate and in the microstructures, administering VEGF (50 ng mL^−1^), TGF-β1 (5 ng mL^−1^) and BSA (0.1%). This was designed as a control sample. Cell culture medium with minimal fetal bovine serum concentration (0.1%) was used in all the experiments to minimize the cell stimulation by the natural presence of such molecules and growth factors. On day 7, samples were fixed, and an immunofluorescence assay was performed before imaging by confocal laser scanning microscopy. The contribution of the microstructures and growth factors was first assessed by observing the cellular organization in the two substrates (flat and 3D) in the presence of VEGF, TGF-β1 and BSA. Cells were immunostained for collagen I to evaluate its specific production, and CD31, to evaluate endothelial cell maturation, while fibroblasts were already fluorescent due to constitutive expression of RFP. The analysis of confocal images was performed considering the central portion of the array (green box in the graphical sketches, Fig. 6), where cells were less perturbed by velocity fields and wall shear stresses present near the inlet and outlet of the bioreactor's chamber. In static co-culture experiments, 7 days of continuous culture were not sufficient for the cells to organize spatially, while they randomly arranged on both flat (Fig. 6A, left panel) and 3D substrates (Fig. 6A, right panel) and in any of the tested chemical stimulations. Experiments under dynamic conditions were performed combining medium perfusion, three-dimensionality (microstructures' array), and chemical stimulation (growth factors). Following the predictions of the computational model, a flow rate (*Q*) of 5 μL min^−1^ was applied by the high precision syringe pump perfusing the bioreactor's chambers, which therefore led to a velocity of approximately 5 μm s^−1^ and a wall shear stress of nearly 0.32 mPa. These values were obtained within the range of the *in vivo* interstitial flow, which was the target to be achieved for stimulating angiogenesis,^43^ and the condition required to allow the bioreactor to mimic *in vivo* perfusion.^34^ Concurrently, by performing medium change and growth factors administration every two days, live co-cultures were continuously monitored, thanks to the optical accessibility of the MOAB. After 5 days of perfusion, the microstructures were densely populated by cells that proliferated under both chemical and physical stimuli. On day 7 of the experiment (corresponding to 2 days of static cell culture before circuit assembly plus 5 days of perfusion), the co-cultures were fixed and stained for imaging by confocal microscopy. While in the absence of 3D microstructures cells did not show any specific spatial organization (Fig. 6B, left panel), cells adhered and proliferated within the microstructures and in between them (Fig. 6B, right panel). As can be noticed, the cells and their nuclei tended to align following the flow direction from the inlet to the outlet, mainly in the areas between the microstructures, creating cellular connections between two consecutive microstructures. The formation of elongated tubule-like structures associated with endothelial cell organization was further investigated by analyzing the CD31 staining signal pattern. The results showed that, after 5 days of interstitial flow, endothelial cells were already aligned along the flow direction, in the presence of both the following stimuli: VEGF administration and 3D microstructures only (Fig. 6B, right panel). Most of the endothelial tubule-like structures connected adjacent microstructures. This phenomenon suggests a preliminary process of vascularization. A quantitative analysis of lengths and diameters was performed to characterize the endothelial tubule-like structures (Fig. S2A†) obtaining an average length of 128.1 ± 8.6 μm and an average diameter of 8.5 ± 2.4 μm, in accordance with the dimensions of *in vivo* dermal capillaries.^46^ Concerning the production of the connective matrix, in terms of collagen I, performed by fibroblasts, under static conditions at day 7 no significant differences were highlighted on flat substrates, between the samples administered with the growth factors and the control sample (Fig. 6C), indicating that cells are not sufficiently stimulated for synthesizing collagen I. On the other hand, in the presence of the microstructures, cells treated with TGF-β1, and VEGF synthesized a significantly different amount of collagen I with respect to the one quantified in not treated cells, thus demonstrating the essential role of the chemical stimulation combined with the presence of a 3D microenvironment (Fig. 6E), indicating a physiological behavior. By analyzing the dynamic conditions, VEGF and TGF-β1 effectively triggered RFP-HDF in synthesizing collagen I, more than if not chemically stimulated, leading to significant differences both on 2D (area surrounding the microstructures, Fig. 6D) and 3D substrates (Fig. 6F). Within the microstructures' array, a predominant effect of TGF-β1 over VEGF administration was appreciable. Therefore, our dynamic system allowed the integration of chemical and physical stimuli to obtain a collagen I matrix supporting the tubular organization of endothelial cells, thus mimicking the physiological angiogenesis process in a dermal perivascular microenvironment. Static experiments were extended up to 13 days and demonstrated the relevant contribution of both the microstructures and VEGF administration. In flat conditions (Fig. 7A), endothelial cells randomly disposed among fibroblasts, without forming any well-organized structures similarly between the untreated and treated samples, with a predominant effect of TGF-β1 over VEGF in terms of collagen I expression (Fig. 7B). On the contrary, in the presence of the microstructures and VEGF stimulation, HUVEC arranged in the shape of a primordial microvascular network, supported by the surrounding connective matrix constituted by fibroblasts (Fig. 7C). As reported in Fig. 7D, quantification of collagen I synthesized by RFP-HDF at day 13 demonstrated that, in the presence of the 3D substrate, significant differences were visible between samples treated with VEGF and TGF-β1 with respect to the control. Upon VEGF administration, branches formed in the presence of the 3D substrate were quantified in terms of number, length, and diameter. The analysis led to counting an average number of 4.3 ± 1.49, an average length of 49.4 ± 17.5 μm, and an average diameter of 6.4 ± 1.8 μm, comparable to what is reported in *in vitro* angiogenesis assays performed up to 14 days.^47,48^ The tubule-like structures' length and diameter were measured considering their full extension, obtaining an average length of 130.6 ± 71.6 μm and an average diameter of 8.5 ± 3.6 μm (Fig. S2B†), in line with results obtained under dynamic conditions at day 7 (Fig. S2A†) and dermal capillaries *in vivo*.^46^ To further corroborate the results, cells grown in the flat area surrounding the microstructures' array were analyzed under static conditions on day 7 (Fig. S3A†) and day 13 (Fig. S3C†). The confocal acquisitions showed that the co-cultures did not follow precise organized patterns and were randomly arranged, generating a consistent tissue-like layer of endothelial cells, fibroblasts and connective tissue matrix. In terms of collagen I synthesis, no significant differences were appreciable between all the analyzed samples (Fig. S3B and D†), and without the presence of a 3D microenvironment, endothelial cells were not triggered to arrange into tubular structures. Interestingly, these results led to the conclusion that the microstructures did not have any apparent paracrine effect on cell behavior, presenting no significant differences between the treated and not treated samples, as well as between the two administered growth factors. Confocal images of co-cultures under dynamic conditions upon administration of VEGF, acquired at 10, 20, 40 μm height from the bottom of the culture chamber (Fig. S4A†), showed that cells grew and organized within the entire volume of the microstructures. Moreover, confocal microscopy orthogonal views and 3D reconstruction clearly showed that tubule-like structures were formed in the 3D microenvironment, occupying a 3D volume (Fig. S4B and C†). Since lumens were not visible from these images, future efforts will be devoted to further characterizing our *in vitro* model in the presence of perfusion. Summarizing the results obtained under static conditions, endothelial cells and fibroblasts stimulated by VEGF and TGF-β1 needed approximately two weeks to organize creating a primordial microvascular network, in the presence of the microstructures and upon VEGF administration only, as previously reported.^13,49^ Static experiments performed on 7 days of continuous culture, as under dynamic conditions, revealed the lack of newly formed tubule-like structures, even with the combination of microstructures and VEGF, highlighting the need for 13 days for cells to organize into tubular structures without perfusion. Tubule-like structures under static conditions at day 13 revealed a comparable average length and diameter values within the range of the ones found in the literature.^48^ It is worth noting that, while under static conditions at day 13 a branched sprouting of HUVEC was observed, the interstitial flow applied under dynamic conditions prompted these cells to connect consecutive microstructures, with tubule-like structures of length and diameter in line with dermal capillaries *in vivo*.^46,50^ The specific role of perfusion in promoting a fast formation of branching microvascular networks *in vitro* has been reported in previous studies, further corroborating our results.^51^ By measuring the full-length extension of tubule-like structures obtained under static culture conditions, values were compatible with the ones obtained under dynamic conditions. This finding further suggested that the presence of an interstitial flow allowed endothelial cells to form vascular sprouts within 7 days instead of the 13 required under static conditions. Concerning the contribution of growth factors to collagen I synthesis under static conditions, TGF-β1 and VEGF led to a higher amount of connective tissue matrix production in 3D samples, both at 7 and 13 days, with respect to the untreated control sample. This indicates that the combination of the mechanical constrains, acted by the microstructures, and the chemical stimulation is fundamental to create the required support for allowing endothelial cell organization. Comparing static and dynamic co-cultures carried out till day 7, tubule-like structures formed only under dynamic conditions, suggesting that perfusion combined with the presence of microstructures is the only variable significantly prompting endothelial cell organization. Under the administration of VEGF and TGF-β1, flat static co-cultures presented the lowest levels of collagen I, thus suggesting that the chemical stimulus given by the growth factors, together with the presence of the 3D substrate and with interstitial flow, led to a higher stimulation of RFP-HDF. After 13 days of culture on flat substrates, only cells treated with the growth factors produced a quantity of collagen I significantly different from the control. In summary, analyzing collagen I synthesis at day 7 and day 13 under static conditions, a higher quantity of collagen I was appreciable at 13 days respect to 7 days, meaning that fibroblasts effectively continued to be stimulated over time. This should be related to the fact that almost two weeks were necessary for endothelial cells to form a primordial microvascular network, supported by a robust presence of surrounding connective tissue. In static co-cultures performed on day 13 and dynamic co-cultures performed on day 7, we observed that collagen I production in the control samples and upon TGF-β1 administration did not reveal any significant difference between the two culture conditions, as well as between the flat and 3D substrates. Generally, TGF-β1 led to a higher collagen I synthesis with respect to control samples, and microstructures and perfusion did not affect its production, while keeping the perivascular microenvironment protected from fibrotic-like reactions, often observed during *in vivo* tissue neovascularization.^52^ This observation was not possible in the case of VEGF administration, where significant differences were found between flat and 3D substrates, both under static and dynamic conditions, thus suggesting that microstructures contributed to VEGF internalization. HUVEC arranged into tubular structures in 13 days in the presence of the microstructures under static conditions, presenting the highest amount of collagen I with respect to 7 days under dynamic conditions and on 2D substrates. Thus, no evident correlations were found between collagen I production and tubule-like structures formation.

## Conclusions

This research work relied on a comprehensive workflow that included both a computational and experimental model of a dermal perivascular microenvironment on a chip. The computational model provided the optimal parameters (flow rate, velocity, and wall shear stress) required to set the dynamic experiments. These aimed at mimicking the angiogenesis process under the influence of a physiological-like interstitial flow. To our knowledge, this is the first 3D dynamic model of angiogenesis, supported by computational predictions of mechanobiological field variables, and involving cellular co-cultures chemically stimulated with growth factors, thus assessing the uniqueness of the model proposed as truly exhaustive in encompassing as many elements as possible and to accurately simulate the *in vivo* environment. Indeed, the most common *in vitro* angiogenesis assays exploit co-cultures of endothelial cells and fibroblasts, because of their crosstalk in generating new vessels, triggered by the effect of administered growth factors, but without combining also three-dimensionality and fluid dynamics.^49,53,54^ The innovation of this work has been the use of the millifluidic bioreactor that allowed the exposure of cells seeded in 3D microstructured substrates to an interstitial flow as a physical stimulus. Moreover, for the first time, apart from perfusing the cellular co-cultures within a 3D microenvironment, the model has included also the use of VEGF and TGF-β1. These growth factors physiologically contribute to angiogenesis *in vivo*^55,56^ by allowing the analysis of three different experimental conditions (control, VEGF and TGF-β1) simultaneously in the three independent culture chambers. Our highly versatile device has been developed with the primary objectives of performing experiments in optically accessible microstructured chambers in the presence of interstitial perfusion, minimizing the required working volumes, and thus significantly reducing the experimental costs. These features make its most immediate translational application in drug testing and in pharmacological settings, reducing and limiting trial costs and animal testing altogether (reduction, refinement, replacement). The scalability to larger tissues for clinical applications is beyond the aim of this work and remains a limitation of the present model, although our promising results could envisage further evolution in the design of the device for modeling multiple tissues in a preclinical context. In conclusion, this work provides an exhaustive and robust *in vitro* model of the dermal perivascular microenvironment. Future experiments will be devoted to investigating the potential of the *in vitro* formed tubule-like structures to be perfused, for proving the presence of continuous lumens.^20^

## Materials and methods

### Sample preparation for 3D microstructure fabrication

A drop (46 μl) of SZ2080 photoresist was deposited on a glass coverslip (Cover Slips, ThermoFisher Scientific, USA) with a diameter of 12 mm and a thickness of 170 μm. The drop cast procedure was performed under a chemical hood, in a room with UV-free light, to protect the sample from any undesired polymerization. SZ2080 photoresist is a hybrid organic–inorganic resin composed of 80% silicon and 20% zirconium with 1% w/v of Irgacure 369 (1-benzyl-1-(dimethylamino)propyl 4-morpholinophenyl ketone, Sigma Aldrich, USA) as a photoinitiator.^57,58^ This material has been previously widely validated, demonstrating its high optical quality, good mechanical stability, biocompatibility and low shrinkage properties.^59–62^

### Two-photon laser polymerization setup and 3D microstructure fabrication

The femtosecond laser source used for fabricating the 3D microstructures was a laboratory-made mode-locked oscillator, based on Yb:KYW in a cavity-dumped configuration. Its characteristic wavelength was 1030 nm, with a repetition rate of 1 MHz, a pulse energy of 1 μJ, a pulse duration of approximately 300 fs and an average maximum output power of approximately 1 W. The setup was composed of optical elements such as mirrors, lenses, shutters, a telescope, a 100× oil immersion objective (Plan-Apochromat, Carl Zeiss, Oberkochen, Germany), a spatial light modulator (SLM, PLUTO NIR-049, HOLOEYE, Germany), a gimbal (Gimbal Mounts 100, Thorlabs, USA), a computer numerical control (CNC) software program (Automation 3200 CNC Operator Interface, Aerotech, USA) and a CMOS camera (DCC1545M, Thorlabs, USA).^36,63^ Upon setup alignment, the deposited glass sample was mounted and fixed on a specific aluminum holder screwed to the gimbal mechanical system used for focusing the laser beam inside the photoresist volume: a brushless motion stage (ANT130XY Series, Aerotech, USA) allowed planar motion of the sample in *XY* directions, whereas an additional stage (ANT130LZS Series, Aerotech, USA) permitted the vertical movement (*Z* direction) of the objective. The laser beam was tightly focused by the high numerical aperture (NA 1.4) 100× oil immersion objective on the photosensitive material, passing through the sample glass substrate. The focus was found using a camera, placed vertically above the optical elements, and connected to a visualization software program (μEye Cockpit, IDS, Germany), through which, switching on a red-light emitting diode placed under the sample holder in the central cavity of the gimbal, the visualization was possible during the writing procedure (Fig. 2A). After the fabrication process, samples were developed to remove all the unpolymerized photoresist under the chemical hood (Fig. 2B). Briefly, the sample was gently placed on a metallic cage structure with the resist side to the top and soaked in a beaker with 10 mL of propan-2-ol and methyl isobutyl ketone (Sigma-Aldrich, USA) in a ratio of 50 : 50 (v/v) for 35 minutes. Then the sample was washed with propan-2-ol and dried with a low flux of nitrogen. Scanning Electron Microscopy (SEM, Phenom Pro, Phenom-World, Netherlands) under high vacuum conditions of 10 kV was then performed.

### Miniaturized optically accessible bioreactor (MOAB) and circuit assembly

The glass coverslips with the fabricated 3D microstructures' array were joined to the MOAB's chambers by sealing it with a biocompatible UV-curable glue (Loctite 3345, Henkel, USA) on top of each chamber's lid, and cured with a diode UV lamp (Fig. 2C), with *λ* = 365 nm (Hamamatsu, Japan). The MOAB was assembled to a hydraulic circuit connected to a high precision syringe pump (Harvard Apparatus, PHD∣Ultra, USA) set to make the cell culture medium flow. Each circuit was composed of a 20 mL Luer lock syringe (InJ/Light®, RAYS S.p.A., Italy) filled with cell culture medium and a silicon tubing system connected to the inlet and the outlet of each chamber. The lid of each reservoir presented two exits: one for the outlet tube and one for a 0.22 μm PES (Primo® Syringe Filter, EuroClone, Italy) to avoid contamination and to balance the internal–external atmosphere. At the outlets, three reservoirs (Primo® EZ tubes 50 mL PP, conical centrifuge tubes, EuroClone, Italy) collected the waste products (Fig. 2I). Two days after cell seeding in the lids, the circuit was assembled, priming with 0.1% EGM medium enriched with the growth factors plus the control was performed and the bioreactor was incubated at 37 °C, 5% CO_2_. Every two days, the syringes were refilled with fresh cell culture medium enriched with the growth factors plus the control under a sterile biological hood till day 5 of perfusion.

### Computational analyses of fluid dynamics and mass transport

A multiphysics computational model (COMSOL Multiphysics® 6.1 software – Stockholm, Sweden) was set and a finite element computational fluid dynamics (CFD) study was carried out together with a transport of diluted species physics for simulating oxygen consumption by the cells (consumption rate = 4 × 10^−17^ mol s^−1^ for HUVEC).^64,65^ The bioreactor's chamber with the array of microstructures was designed (SolidWorks® 2023 – Dassault Systèmes, France), starting from the extrusion of a 6 mm × 3 mm × 0.5 mm rectangle. The inlet and outlet were modeled with two cylinders of 0.76 mm diameter, 3 mm high and the microstructures were placed inside the chamber as two rows of 8 each, positioned at half of the chamber's width and equally spaced along the chamber's length. The longitudinal symmetry of the geometry was exploited to perform the simulations, thus considering half of the entire model, and reducing the computational load. A free tetrahedral mesh, composed of 12.885.976 domain elements, 1.497.898 boundary elements, and 387.370 edge elements, was built to perform the simulations, setting a normal element size. A 3D stationary study was used to evaluate the velocities, and the wall shear stresses inside the chamber, considering the flow as fully developed at the entrance of the chamber. The cell culture medium was approximated to water at 37 °C, and laminar flow physics was considered. The following boundary conditions were imposed: i) at the inlet, a velocity *u* equal to *Q*/*A*_in_ was defined, where *Q* was the flow rate, *A*_in_ was the cross-section area of the inlet tube, ii) at the outlet, the pressure, *p*, was set at 0 Pa to avoid backflow, iii) along the *X* axis, the symmetry plane was selected, iv) on the walls, the no slip condition was set. The parametric sweep regarding the flow rate was functional to the evaluation of the best value of *Q* that could generate velocities and shear stresses within the physiological ranges. Since the more significant velocity variations were computed along the *Z* axis, with respect to the *Y* one, just one component of shear stress was considered, such as *τ*_*zx*_, calculated as *μ*d*u*_*x*_/d*z*. Punctual velocity measurements were made in correspondence to the center of the first, the fifth and the tenth pores inside the first, the fourth and the eighth microstructure starting from the inlet of the chamber, at three different heights, 10, 20 and 40 μm. The wall shear stresses were measured averaging the values obtained along the three beams, on the side towards the outlet, of each pore considered (the first, the fifth and the tenth of the same microstructure), at 10, 20 and 40 μm height. The simulations for evaluating the oxygen concentration profile were performed setting 50.000 cells inside the chamber, considering cells at confluency. Since, during the sample seeding, the drop of cell suspension was placed on the array of microstructures, most of the cells (32.000) populated the array, whereas the remaining 18.000 adhered to the flat substrate on the side of the microstructures; as aforementioned, fibroblasts were not considered in the model. The initial oxygen concentration was set at 0.2 mol m^−3^, according to Henry's law and the oxygen solubility in the cell culture medium. The diffusion coefficient for the oxygen in the medium was set at 2 × 10^−9^ m^2^ s^−1^.^66^ The Michaelis–Menten kinetics was used to model the consumption of oxygen by HUVEC: in the case of cells inside the microstructures a volumetric oxygen consumption was modelled, whereas for the cells on the flat bottom of the chamber, an outgoing superficial flow was modelled. The following parameters were used for the Michaelis–Menten kinetics: i) *V*_max_ = 4 × 10^−17^ mol s^−1^, the maximum reaction rate approached by the system at saturating oxygen concentration,^64^ ii) *K*_m_ = 5.5 × 10^−4^ mol m^−3^, defined as the concentration of oxygen at which the reaction rate was half of *V*_max_.^67^ The oxygen concentration profile was evaluated along a line at a height of 10 μm from the bottom of the chamber, crossing the microstructures' array, from the inlet to the outlet, and deriving the oxygen drop associated with a specific input flow rate.

### Cell co-cultures under static and dynamic conditions

Human umbilical vein endothelial cells, HUVEC (00191027, Lonza Bioscience, Switzerland), and RFP-HDF Red Fluorescent Human Dermal Fibroblasts (P20204, Red TTFLUOR HDF, Innoprot, Spain), expressing TurboFP602, a red-shifted variant of the red fluorescent protein TurboRFP from sea anemone *Entacmaea quadricolor*,^68^ were used at passages 1–7 and maintained in T75 cell culture flasks (Biofil). HUVEC were cultured in endothelial growth medium (EGM, ATCC Primary Cell Solutions), supplemented with 2% fetal bovine serum (FBS, EuroClone, Italy), 1% penicillin/streptomycin, 1% l-glutamine (EuroClone, Italy) and 1% endothelial cell growth supplement (ECGS, Innoprot, Spain) and RFP-HDF in Dulbecco's modified Eagle's medium (DMEM, EuroClone, Italy), supplemented with 10% FBS and 1% penicillin/streptomycin (EuroClone, Italy) at 37 °C, 5% CO_2_. Before seeding, cells were trypsinized with 1 mL of 0.1% trypsin/EDTA solution (EuroClone, Italy) and counted. A cell suspension was obtained composed of both the cell lines at a ratio of 5 : 1 (HUVEC : RFP-HDF)^25,69,70^ in complete EGM medium. The choice of using this medium relied on the fact that HUVEC constituted 80% of the cell co-culture population and that they were more susceptible to changing cell culture conditions than fibroblasts. For the static experiments, glass coverslips (Cover Slips, ThermoFisher Scientific, USA) with a diameter of 12 mm, with or without (flat control) the fabricated array of microstructures, were sterilized with 100% ethanol and UV irradiation for 10 minutes under a sterile biological hood and placed in a 24-well plate (EuroClone, Italy). Before seeding, samples were incubated with 50 μL of 15 μg mL^−1^ fibronectin (PromoCell, Germany), for 2 hours at 37 °C 5% CO_2_, to promote cellular adhesion and, after removal, the glass coverslips were left dry overnight under a sterile biological hood. A total amount of 50.000 cells per sample was plated in a 50 μL medium drop. Cells were let adhere for 2 hours, and then 450 μL of complete EGM were added to each well. Cell co-cultures were then administered with 5 ng mL^−1^ TGF-β1 (100-21, Recombinant Human Transforming Growth Factor β1, PeproTech, USA), 50ng mL^−1^ VEGF (100-20, Recombinant Human Vascular Endothelial Growth Factor_165_, PeproTech, USA), and 0.1% bovine serum albumin (BSA, Sigma Aldrich), the growth factors' diluent buffer as a control, in EGM 0.1% FBS. The concentrations of TGF-β1 (5 ng mL^−1^) and VEGF (50 ng mL^−1^) were chosen based on the supporting literature. Concerning TGF-β1, Chen *et al.* used 5 ng mL^−1^ of TGF-β1 for stimulating myofibroblast differentiation and collagen upregulation;^71^ in a study by Luo *et al.*, fibroblasts were stimulated with 5 ng ml^−1^ TGF-β1 for inducing proliferation, migration and collagen I synthesis;^72^ and Kang *et al.* stimulated fibroblasts with 5 ng ml^−1^ TGF-β1 observing increased levels of collagen I and its activation to release immunomodulatory PD-L1 in extracellular vesicles.^73^ Concerning VEGF, in a study by Vickerman *et al.*, 50 ng ml^−1^ VEGF were administered to endothelial cells for promoting capillary morphogenesis, showing efficient cellular organization.^20^ Whisler *et al.* stimulated endothelial cells by using 50 ng ml^−1^ VEGF to enhance vessel sprouting and generate vascular networks.^74^ Sturtzel *et al.* demonstrated that endothelial cell proliferation was induced by 50 ng ml^−1^ VEGF in a sprouting assay for mimicking angiogenesis and the same concentration was used for performing tube formation and migration assays with endothelial cells by Wang *et al.*^75,76^ The medium was changed every two days. The choice of using EGM at minimum serum concentration (0.1%) was functional to investigate the pure effect of growth factors on cellular behavior. Static co-cultures were kept at 37 °C, 5% CO_2_ up to day 7 and day 13. For the dynamic experiments, the same procedure was performed in the three chambers' lids. Before cell seeding, the bioreactor's chambers were pre-incubated with 50 μL of 15 μg mL^−1^ fibronectin (PromoCell, Germany), for 2 hours at 37 °C, 5% CO_2_, to promote cellular adhesion and, after removal, the chambers' lids were left to dry overnight under sterile conditions. A 50 μL medium drop with 10.000 cells at a ratio 5 : 1 (HUVEC : RFP-HDF) in complete EGM medium was deposited in each chamber and the lids were placed inside a 50 mm Petri dish, housed in a 150 mm plate with 5 mL of PBS, to avoid the long-term evaporation of the drop. Cells were incubated for two days under static conditions, then the MOAB was assembled, culturing cells in 0.1% EGM enriched with the same concentrations of growth factors as for the static experiments. Under dynamic conditions, co-cultures were perfused for 5 days.

### Immunofluorescence assay and confocal laser scanning microscopy imaging

At day 7 and day 13, co-cultures under static and dynamic conditions were rinsed twice in PBS and fixed with paraformaldehyde solution (w/v 4%) for 10 min, before performing staining of the nuclei, collagen type I and CD31. Samples were then washed three times with glycine 0.1 M solubilized in PBS and permeabilized with 0.25% Triton-X-100 in PBS for 10 min. To block nonspecific binding of the antibodies, samples were incubated with a solution of 2% bovine serum albumin (BSA) and 0.1% Tween in PBS for 4 hours at room temperature. Fibroblasts were visible since they constitutively expressed TurboFP602, a red-shifted variant of the red fluorescent protein. Anti-CD31 (BS-0468R, dilution 1 : 200, Bioss Antibodies, USA) and anti-rabbit 647 (AF647, dilution 1 : 1000, ThermoFisher Scientific, USA) were used to stain endothelial cells, specifically their membrane.^77^ Anti-collagen I (MA1-26771, dilution 1 : 2000, ThermoFisher Scientific, USA) and anti-mouse 488 (AF488, dilution 1 : 1000, ThermoFisher Scientific, USA) were employed to stain collagen type I. Primary antibodies were incubated overnight at 4 °C, then the samples were rinsed three times in 0.1% Tween in PBS before incubation with the secondary antibodies in a solution of 2% BSA 0.1% Tween in PBS (45 minutes). After washing three times with 0.1% Tween in PBS, cell nuclei were stained with 1 μM Hoechst 33342 (ThermoFisher Scientific, USA). Finally, samples were washed with PBS and distilled water (dH_2_O) and mounted with 10 μL MOWIOL^®^ 4-88 Reagent (Sigma-Aldrich, USA). Confocal images were acquired (Fluoview FV10i, Olympus, Japan) and then analyzed through ImageJ software (1.53, NIH, USA), selecting the ROI (region of interest) suitable to fit a pore of the microstructure (50 × 50 μm^2^). Specific ROIs were created inside each 50 × 50 μm^2^ pore for evaluating collagen I fluorescence intensity, thus excluding the contribution due to SZ2080 autofluorescence. The fluorescence intensity of collagen I synthesized by fibroblasts was evaluated within and on the side of the array, plus on totally flat glass coverslips as a control. Fluorescence analysis was based on the quantification of the mean gray value by selecting ROIs fitting a pore of the microstructure and analyzed through ImageJ software (1.53, NIH, USA). Measurements of the length and diameter of the tubule-like structures formed by endothelial cells were carried out. The analysis of the samples under static conditions was carried out measuring lengths and diameters of the branches, together with the full-length and diameters of the tubule-like structures. The same measurements were performed on samples obtained under dynamic conditions. Z-stack images were acquired within the height of the entire microstructure (40 μm) to evaluate the disposition of the cells inside and outside the array and compared to a flat substrate. The statistical analysis of the collected data was performed with OriginPro (OriginPro 2024b software, OriginLab Corporation, Northampton, Massachusetts, USA) applying non parametric tests with Kruskal–Wallis ANOVA with Dunn's multiple comparison test for not normal data and ordinary one-way ANOVA with Tukey's mean comparison test for normal data, with the following significance: *p-value < 0.05; **p-value < 0.01; ***p-value < 0.001; ****p-value < 0.0001.

## Data availability

The data supporting this article have been included as part of the ESI.†

## Author contributions

CM, EJ, and MTR designed experiments and supervised the study. CM, AB, and SM wrote the paper. CM and SM performed experiments, collected and analyzed the data. AB, SM, and TB performed computational studies and analyzed the data. GB contributed to supporting *in vitro* experiment execution. SP contributed to perfusion experiments design and setting up. GCe and RO developed the two-photon polymerization process and provided the laboratories to perform the microstructure fabrication. CC and LC contributed to the array design and to the setup and optimization of the two-photon polymerization process. GCh, EJ, and MTR revised the manuscript. All authors contributed to this article and approved the submitted version.

## Conflicts of interest

MTR, RO, and GCe are founders of the university spinoff company MOAB S.r.l. and hold shares. The other authors declare no competing financial interest.

## Supplementary Material

LC-025-D4LC00898G-s001
