# Supplementary material for: A 3D millifluidic model of a dermal perivascular microenvironment on a chip
Source: Lab Chip. 2025 Jan 6;25(3):423–39. doi: 10.1039/d4lc00898g (PMC11701800; doi:10.1039/d4lc00898g)
Supplement: LC-025-D4LC00898G-s001 [file LC-025-D4LC00898G-s001.pdf]

Supplementary Information (SI) for Lab on a Chip.
This journal is © The Royal Society of Chemistry 2025

A

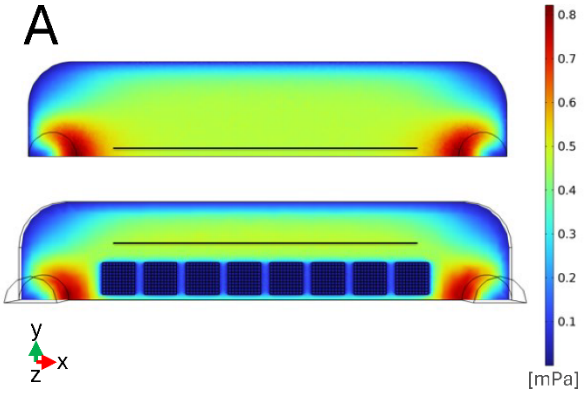

B

Flow rate	WSS flat MOAB	WSS around microstructures
5µl/min	0.4629 mPa	0.4603 mPa

Figure S1. Wall shear stress measurement. A) Wall shear stress measurement, at 10 µm from the bottom of the chamber, within a totally flat MOAB chamber and in the flat area surrounding the microstructures' array in a fabricated chamber's lid. The black lines indicate where the measurements were performed. B) Table summarizing the average values of wall shear stress (WSS) obtained in a totally flat MOAB chamber and in the flat area surrounding the microstructures' array, setting a flow rate of 5 µL min^{-1}.

A

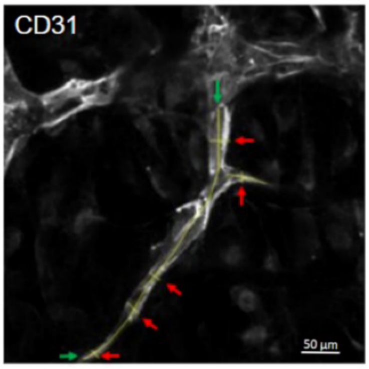

B

Culture	Average length (Branches)	Average diameter (Branches)	Number (Branches)	Average length (tubules)	Average diameter (tubules)
Static	49.4 ± 17.5	6.4 ± 1.8	4.3 ± 1.49	130.6 ± 71.6	8.5 ± 3.6
Dynamic	-	-	-	128.1 ± 8.6	8.5 ± 2.4

Figure S2. Branches and tubules measurement. A) CD31 staining of static co-cultures at day 13. Red arrows indicate the measured diameters, green arrows highlight the measured lengths. B) Table summarizing the average length, diameter and number of branches and average length and diameter of tubules measured in static co-cultures at day 13 and in dynamic co-cultures at day 7. Measurements are expressed in micrometers (µm).

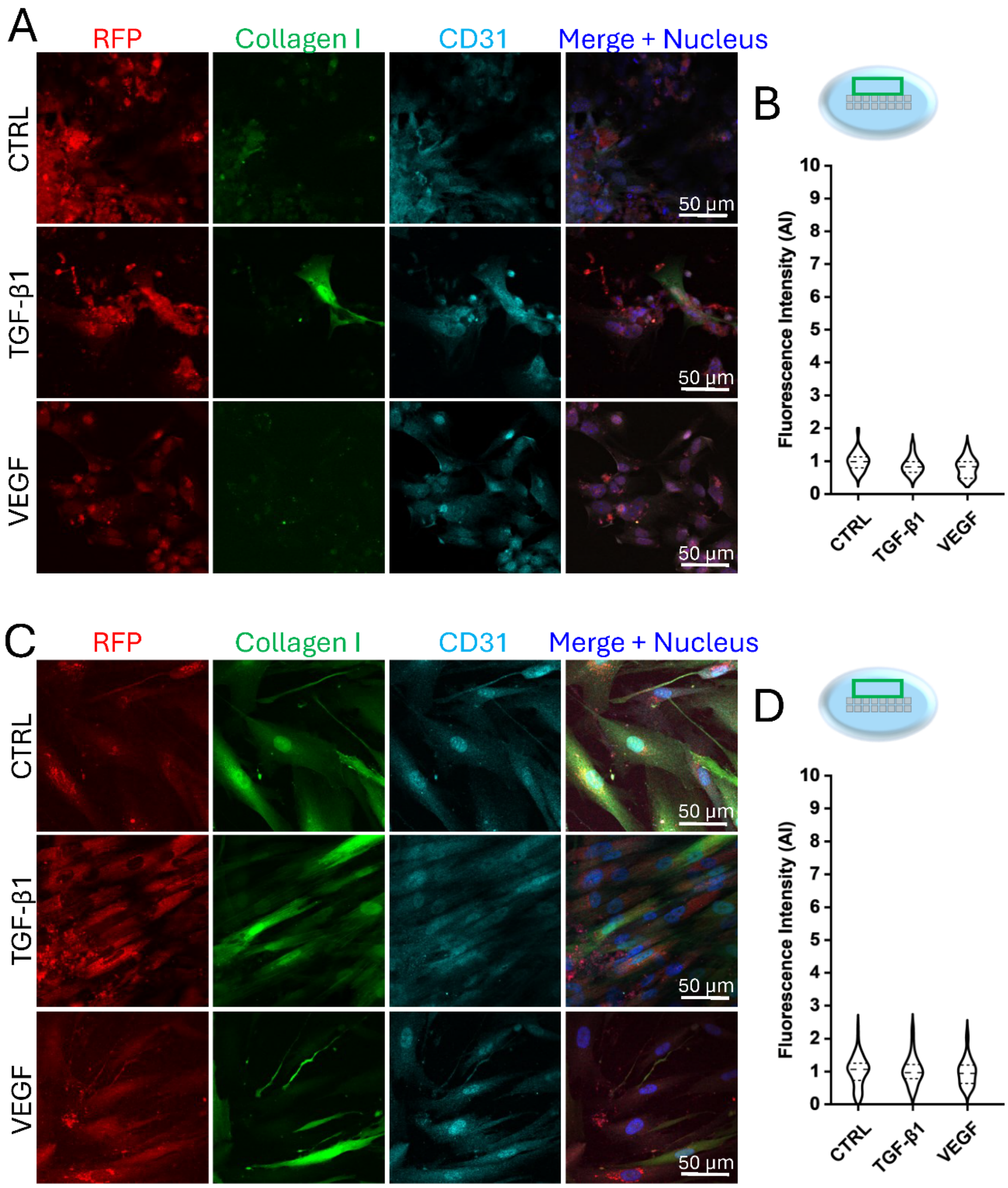

Figure S3. Immunofluorescence and collagen I fluorescence intensity quantification of static co-cultures at day 7 and day 13. A) Confocal images of the stained static co-cultures at day 7 on the flat area surrounding the microstructures. RFP, collagen I and CD31 are visible in red, green and cyan, respectively. B) Quantification of collagen I synthetized by RFP-HDF at day 7 on the flat area surrounding the microstructures in static experiments in presence of VEGF or TGF-β1. Data are normalized with respect to the control (0.1% BSA), n ≥ 27 for each condition tested. C) Confocal images of the stained static co-cultures at day 13 on the flat area surrounding the microstructures. RFP, collagen I and CD31 are visible in red, green and cyan, respectively. D) Quantification of collagen I synthetized by RFP-HDF at day 13 on the flat area surrounding the microstructures in static experiments, in presence of VEGF or TGF-β1. Data are normalized respect to the control (0.1% BSA), n ≥ 27 for each condition tested. The green box in the graphical sketches (not scaled) above images indicates regions of the samples where acquisitions were performed.

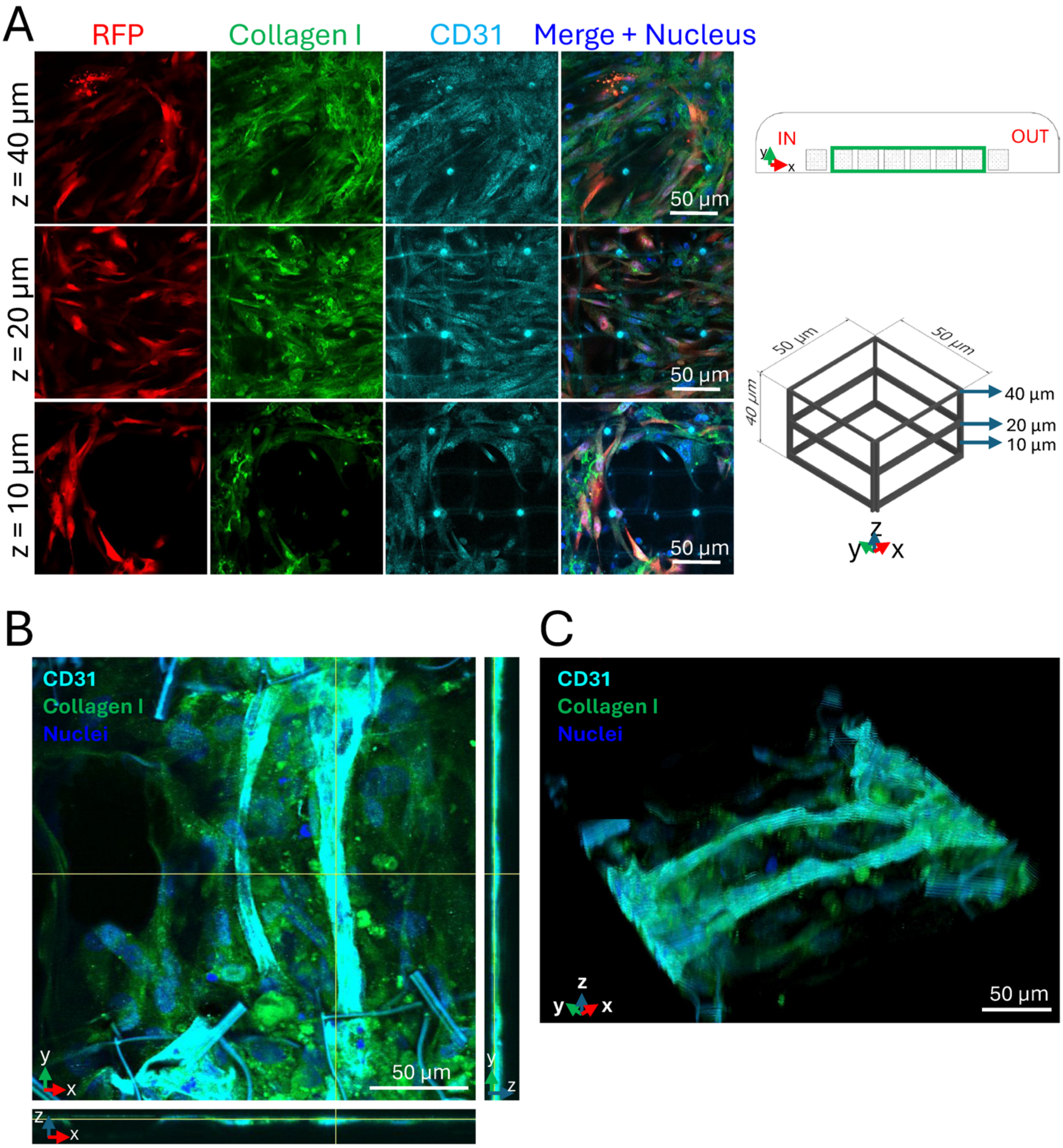

Figure S4. Immunofluorescence of dynamic co-cultures at day 7 in the microstructures. A) Confocal images of the stained dynamic co-cultures at day 7 upon VEGF administration acquired at 10, 20, 40 µm height from the bottom of the culture chamber. RFP, collagen I, and CD31 are visible in red, green and cyan, respectively. B) Confocal microscopy orthogonal views. C) Confocal microscopy 3D reconstruction. Collagen I and CD31 are visible in green and cyan, respectively.
